# Parallel Genetic Algorithms’ Implementation Using a Scalable Concurrent Operation in Python †

**DOI:** 10.3390/s22062389

**Published:** 2022-03-20

**Authors:** Vladislav Skorpil, Vaclav Oujezsky

**Affiliations:** Department of Telecommunications, Brno University of Technology, Technicka 3082/12, 616 00 Brno, Czech Republic; oujezsky@vut.cz

**Keywords:** Master–Slave, Coarse-Grained, Fine-Grained, parallelized genetic algorithms, SCOOP

## Abstract

This paper presents an implementation of the parallelization of genetic algorithms. Three models of parallelized genetic algorithms are presented, namely the Master–Slave genetic algorithm, the Coarse-Grained genetic algorithm, and the Fine-Grained genetic algorithm. Furthermore, these models are compared with the basic serial genetic algorithm model. Four modules, Multiprocessing, Celery, PyCSP, and Scalable Concurrent Operation in Python, were investigated among the many parallelization options in Python. The Scalable Concurrent Operation in Python was selected as the most favorable option, so the models were implemented using the Python programming language, RabbitMQ, and SCOOP. Based on the implementation results and testing performed, a comparison of the hardware utilization of each deployed model is provided. The results’ implementation using SCOOP was investigated from three aspects. The first aspect was the parallelization and integration of the SCOOP module into the resulting Python module. The second was the communication within the genetic algorithm topology. The third aspect was the performance of the parallel genetic algorithm model depending on the hardware.

## 1. Introduction

Our research involved designing and implementing parallel processing genetic algorithms (GAs). Genetic algorithms are a class of modern algorithms inspired by nature, referred to as evolutionary algorithms. The way these algorithms work predisposes them to parallel processing. Parallelization is a popular method of speeding up not only genetic algorithms. However, genetic algorithms provide several methods of parallelization, which bring additional advantages and disadvantages for solving optimization problems. This paper was based on [1], and its goal was to describe our research in the area of the parallelization of genetic algorithms and the subsequent implementation. Some parts of our research have been gradually published in conference proceedings [2,3,4], and this journal article is a significantly extended version (approximately 70%) of the conference proceedings article [5]. Here, the latest findings and completion of the whole issue are described.

Python was selected as the implementation programming language for the genetic algorithm, so the design was implemented with this language in mind. The proposal, therefore, also includes an overview of the different parallelization methods.

Algorithm parallelization is a valuable tool for making an algorithm more efficient and faster. GAs are very suitable for a large set of problems, but some of them require a more significant amount of time, and therefore, GAs became unusable for them. The most time-consuming operation within GAs is the fitness function evaluation. This function is performed for each individual and is independent of the others. This makes it suitable for parallel processing. Genetic operators: mutation and crossover can work in isolation as they act on one or two individuals. These operators are usually much more straightforward than fitness functions, but can consume more time than calculating a fitness function depending on the crossover/mutation operation. Communication is also a problem for another genetic operator, selection, which often needs information about the entire population. Therefore, we were concerned only with parallelizing the fitness function.

In addition to defining the topology or architecture, the individual models were implemented on different (hardware) architectures. We use terms such as the “node” of a topology or architecture. Thus, a node can be a single point of the logical architecture, i.e., a model point, or a point of the physical architecture, i.e., a computational unit (e.g., a processor or a computer).

The main contribution of this paper is a comparison of the performance of the Master–Slave, Coarse-Grained, and Fine-Grained parallel genetic algorithms using the scalable concurrent operation in Python. This paper completes our research on parallel genetic algorithms’ implementation using the scalable current operation in Python. We give new details not published in [2,3,4,5]. The source code supporting this paper is available at [6].

## 2. The State-of-the-Art

The Master–Slave model works with only one population in the same way as the Serial model, but it processes the fitness function differently. Further processing, if the data of the last not-yet-evaluated individual in the population have already been sent, divides the Master–Slave GA into two types: synchronous, in which the primary (master) node will start sending data from the new population only after the previous one has been processed on all secondary (slave) nodes; asynchronous, when data of all individuals from the population are already sent to the secondary nodes, the primary node starts sending data from the new population to free nodes. The synchronous Master–Slave GA functions the same as ordinary GA, but is faster. Asynchronous systems work differently and require a different approach when modifying ordinary GAs to asynchronous ones. Only the synchronous variant was considered in the GA Master–Slave model.

The number of nodes may be constant throughout the GA calculation or may change over time. In a multi-user environment, proper load balancing among processors would increase the efficiency of both the GA and the system. This type of GA gains efficiency when we have a larger number of processors and a population of a constant size [4].

In the case of fewer processors, the algorithm loses its efficiency, and this is due to the existence of the master node and the related communication between it and the slave nodes. Nevertheless, deploying a load-balancing system can restore the algorithm to efficiency. The most significant advantage of this GA is that it does not change the functionality of the regular GA, and it is therefore effortless for users to modify their regular GA and apply the Master–Slave architecture to it.

The Fine-Grained model works with one global population spatially dispersed into nodes, creating thus a topology with neighborhoods. The closest environment of the node gives the neighborhood, and the neighborhoods may overlap [2]. On the other hand, due to the relative isolation of a neighborhood, the best individuals do not spread as fast as in other types of GAs, which increases the population diversity. Neighborhoods cover the entire node topology and can have different shapes. Different node topologies and their neighborhoods imply different GA behaviors.

Neighborhoods enclose the space where selection can take place. The selection is thus local and parallel (by neighborhoods) compared to other types of GAs. Each individual participates in the selection process only within the neighborhoods of which it is a part (if an individual is a part of five neighborhoods, it will go through five selection processes). As mentioned, only one central element is modified (by crossover and mutation) within one neighborhood.

Frequently used topologies are one- and two-dimensional grids, which are also often used to deploy the computational elements of parallel computers, which is also why they use the GA [7]. However, the use of the bus topology [8] is excluded. When designing a topology, borders are often a problem. Since we want all nodes to be linked to the same number of nodes, we need to ensure that the neighboring nodes (for example, in the case of a square topology, the nodes located on the perimeter of the square) are connected. The curvature of space most often solves this. The line becomes a circle, and the two-dimensional grid becomes a toroid (a body in space, obtained by rotating a closed planar curve about an axis lying in the plane of the curve and not intersecting the curve).

The type of topology also affects the neighborhood schedule. All GA operators are performed in parallel but differently from ordinary GA. The selection is applied only locally. On the other hand, other types of GAs utilize global, centralized, and sequential selection, which requires collecting large amounts of data, leading to communication problems (“bottleneck”). The mutation is independent of other individuals so that it can be performed on each node separately without communication between them. As an operator over two individuals, the crossover already requires communication, the rate of which depends on the population density and the selection algorithm.

However, different behaviors of genetic operators affect the algorithm’s behavior. Therefore, this type of GA may not work on a particular problem in the same way as the ordinary GA. The advantage of efficiency outweighs this disadvantage, with a flexible and scalable implementation in hardware [8]. System nodes are simple and uniform, and their communication is local and at regular intervals. The number of individuals per node must be considered when designing the system. A higher number of individuals would speed up the algorithm, but on the other hand, it would increase the system’s complexity. When adding individuals to nodes, we have to consider increased requirements on the memory, the processor performance, and the communication infrastructure.

The Coarse-Grained model was described in [4]. The main difference between Fine-Grained and Coarse-Grained genetic algorithms is that the Fine-Grained genetic algorithm works with one global population that is spatially dispersed into a large number of nodes, so it is fine-grained. All nodes are identical and contain only one or two individuals. The number of nodes is much larger than for other parallel genetic algorithms. Coarse-Grained genetic algorithms are often referred to as “distributed” and work on multiple populations or “demes”. The process of evolution takes place over each “deme” asynchronously and relatively in isolation. This relative isolation, which may be partially disrupted by migration, is characteristic of the Coarse-Grained genetic algorithm. Determining whether it is a Fine-Grained or Coarse-Grained model is possible by comparing the number of nodes and the number of individuals in one of them. The Coarse-Grained model is when the number of nodes is less than the number of individuals in one and vice versa.

Comparing their space search and speed is a difficult task. A summary of various studies in [7] gave different comparisons of these models while noting that the comparison cannot be absolute, but must be made concerning the particular optimization problem since some studies indicate that the Fine-Grained model is better, while some indicate the opposite. However, the presented theoretical study concluded that with a sufficient number of processors, the Fine-Grained model was faster (regardless of the population size). However, communication and memory requirements were not taken into account.

Each model offers different implementation options. Topology, population size (as well as the neighborhood in Fine-Grained models), the implementation of genetic operators, and migration may vary. Usually, synchronous migration is applied, which occurs at the same predetermined intervals. Asynchronous migration is triggered by some event. An example is an implementation where the algorithm stops migration if it is already close to convergence. Another possible example may be the opposite, where migration begins only when the population is wholly converged (thus restoring population diversity). This raises the critical question of when is it right to migrate, how many individuals should be migrated, and what happens to individuals who will suddenly become redundant in the new population (if a constant number of individuals in the population is used). In general, however, too high or too low a migration frequency can have a negative impact on the quality of the solution. However, it is clear from the overviews of the various models that models with migration generally perform better than those without it.

Different topologies are optimal for different optimization problems. In the Coarse-Grained model, some studies even suggested that the topology shape is not very important if the topology is densely interconnected and not too large, which would cause insufficient “mixing” among individuals. Generally, however, smaller topologies converge faster, but at the risk of lower-quality solutions. Finding the right size is thus an essential step of the implementation.

The parallelization of evolutionary processes is more related to the real processes of evolution that can be observed in nature. Maximum utilization of parallelization is conditioned by its implementation on parallelized hardware. Although implementing the Fine-Grained or Coarse-Grained models on sequential computing units does not bring much acceleration, it is recommended to use it instead of ordinary GA.

The design of a parallel calculation includes, among other things, sending tasks to workstations and also sending the results of calculations back to the source station. However, parallel GA models require communication during the calculation. This includes sending individuals for migration for the Coarse-Grained model and exchanging data for selection, mutation, and crossing within the neighborhood for the Fine-Grained model. Each model contains several nodes interconnected in a certain way, defined by the model’s topology. Each node can also be represented as one role, so it is necessary to design a system that will implement the communication topology of the given model.

A frequently used module for parallel processing of tasks and the creation of communication topologies is mpi4py [9]. This module has been successfully tested in the described research, but it is challenging to perform distributed tasks on multiple workstations. Perhaps the only instruction for distributed processing, that in [10] is very complex. For example, it assumes the creation of shared directories via the Network File System (NFS). Due to its complexity, this module was not selected for the communication design.

Another option when designing the communication is to use some of the Advanced Message Queuing Protocol (AMQP). RabbitMQ already was chosen [9] from several options. According to the official website [11], it is the most widely used “message broker” system with more than 35,000 applications worldwide. The described research was used in the design of the parallel computation and the design of the communication topology.

## 3. Python Modules and Proposal of the Communication Design Using RabbitMQ

Python modules for implementing GA parallelization within Python and their detailed comparison were given in [4]. For clarity, the following is a brief summary of the selected modules. Four selected modules were chosen based on [9,12]. These modules were Scalable Concurrent Operations in Python (SCOOP), Celery [13], RabbitMQ, and Postgresql. We also tested other modules for implementing GA parallelization within Python described in [4], and these were Multiprocessing [14], PyCSP, Pyro4, Dispy, Rpyc, and Disco.

The Celery module provides an asynchronous queue to insert jobs by sending messages. Celery supports RabbitMQ (recommended), Redis, and Amazon Simple Queue Service (SQS) and focuses on real-time operations, and it also supports task scheduling.

SCOOP [15] primarily focuses on scientific computing [9] and provides task parallelization on heterogeneous nodes. The module provides a futures class. This class provides the map, map_as_completed, and mapReduce functions. All three mentioned functions are parallel versions of the map function, which is part of the Python standard library. The SCOOP module [15] is widely used for evolutionary algorithms, Monte Carlo simulations, data mining, and graph traversal problems, for example.

The test scenario for module evaluation contained two workstations running two Python programs inspired by the tutorial [16] and was extended to execute the task on two workstations. The test scenario for the Celery module was inspired by [9,17], and processing by worker units on multiple workstations in the network was added using the message broker RabbitMQ and the backend Postgresql. The Python module pika [18] was used to test the communication between jobs. To store the results, we used the SQLAlchemy module, which also supports the Postgresql database.

RabbitMQ is written in the Erlang language and is based on the Open Telecom Platform (OTP) [9]. The installation, according to the official documentation on the web [11], is simple, but assumes installed support for the Erlang language. It supports the Linux (Debian, Ubuntu, RHEL, CentOS, Fedora, and Solaris), Windows, and macOS operating systems. In our case, it was installed on the Ubuntu and Fedora systems.

The installation is required on only one node because the system is centralized. It is sufficient to install just the Python pika module on other nodes. It provides a connection object that represents the connection to the central node. The required parameters are: Internet Protocol (IP) address and login details. It then provides a Channel object that provides a queue creation (using queue_declare) and exchanges (using exchang_declare). It also provides basic_publish for sending messages and basic_consume for receiving them.

In the official terminology, the sender of messages is referred to as the producer and the recipient as the consumer. Sent messages are queued before being sent. However, the producer does not send messages directly to the queue, but exchanges. Exchanges receive messages from producers and then queue them. There are several types of sorting, such as direct sorting to all queues without distinction or sorting based on topics. The producer thus defines the type of exchange and the queuing method at the beginning of the communication. The consumer then defines from which queues it wants to receive messages.

**Figure 1:**
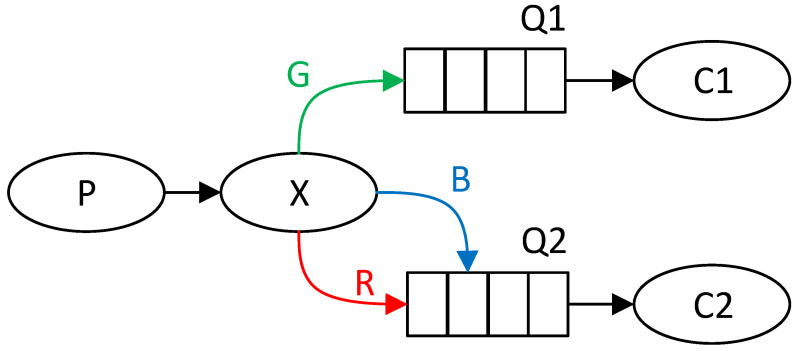
Simplified RabbitMQ model.

A simple example might be sending messages between one producer and two consumers by direct queuing. The first consumer only wants to receive green (G) messages and the second consumer only blue (B) and red (R). The exchange will thus sort messages based on the color into two queues, each for one consumer. This scenario can be seen in Figure 1, where the producer is marked with the letter P, the consumer with the letter C, the queue with the letter Q, and the exchange with the letter X.

Queuing messages can be used to implement the communication topology of the parallel GA model. For a Fine-Grained model, each node would receive messages from the individual neighborhoods of which it is a part. Thus, messages would be queued based on neighborhoods, and each node would only receive messages from specific queues. Furthermore, the node would send messages only to those queues that represent the neighborhood of which it is a part. For a Coarse-Grained model, each strain would communicate through exchanges only with neighboring strains.

The test scenario of this system was implemented on three workstations. RabbitMQ and one of the consumers were running on one of them. The other two launched a producer and another consumer. All the mentioned types of exchanges were tested. A scenario that simulated the Remote Procedure Call (RPC) behavior was also tested. The data sent in the messages were objects serialized according to the JavaScript Object Notation (JSON) standard. The Python module JSON was used for this. All scenarios were taken over and modified from official documentation on the web [11].

When evaluating existing solutions, we can mention the Genetic Algorithm Framework (GAFT) module in Python, which provides, in addition to the standard GA calculation, the possibility of parallelization using Message Passing Interface (MPI) modules (MPICH or OpenMP) [19]. Another interesting module is the Distributed Evolutionary Algorithms in Python (DEAP) [20]. This module provides parallelization using the Scalable Concurrent Operation in Python (SCOOP) module described in [4]. It provides many functions, but the implementation of one of the parallel models is already up to the user.

## 4. Implementation of the Python Module

GA parallelization was implemented using the functions provided by the SCOOP module. In particular, they were the Python class ScoopApp and the map function from the SCOOP futures submodules. The ScoopApp class creates parallel processes on one or more workstations. The user defines the number of processes and workstations on which the processes are to run. Each process runs a Python program on a given path in the workstation’s directory structure. The path to the Python file that contains the program is also user-defined.

The RabbitMQ server supports the implementation of the communication between processes. The connection to the server was made using the Python module pika. The implementation was realized using implementation examples on the official documentation page of the pika module [18,21]. Creating a thread generally ensures asynchrony in programming. Its use is widespread, especially when programming applications that communicate over a network. In this case, the GA calculation and the communication between the processes ran in parallel and independently.

The implementation of the parallel models was divided into two parts. The first was the implementation of the algorithm itself, and the second was the implementation of running the algorithm.

**Figure 2:**
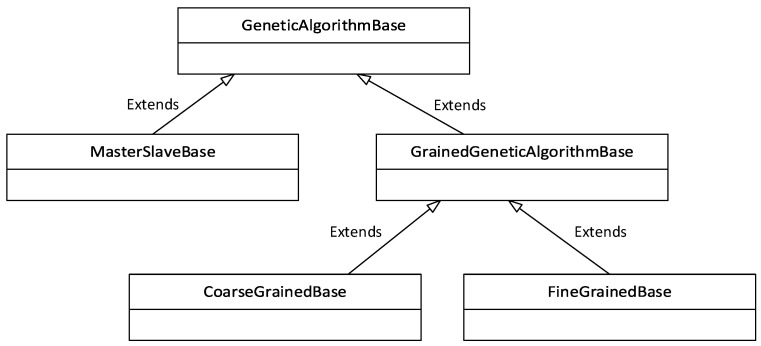
UML diagram of the structure of GA classes.

The implementation of the algorithm was located in the geneticAlgorithms submodule, and its structure is illustrated by the Unified Modeling Language (UML) diagram in Figure 2. The main Python classes were the classes MasterSlaveBase, CoarseGrainedBase, and FineGrainedBase. The auxiliary classes were GeneticAlgorithmBase and GrainedGeneticAlgorithmBase. The word “base” in the class name indicates that it is a base class and can be further modified by users. The GeneticAlgorithmBase class provides the essential functions of the GA: random population initialization, random chromosome generation, crossing, mutation, and pseudorandom selection of individuals based on individual ratings. The GrainedGeneticAlgorithmBase class, in turn, provides common functions for the Fine-Grained and Coarse-Grained module, such as creating and closing a connection to the RabbitMQ server using a Messenger object.

**Figure 3:**
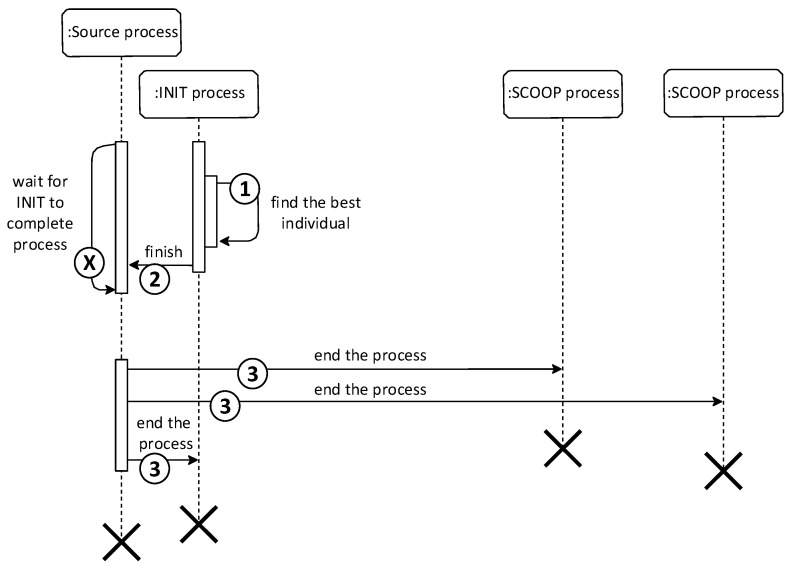
Sequence Master–Slave module termination diagram for 3 nodes.

The implementation of the Master–Slave model algorithm is represented by the Python class MasterSlaveBase. It inherits functionality from the GeneticAlgorithmBase class and then copies the course of the classic GA in the order:

1. Evaluation of individuals;
2. Pseudorandom selection of individuals for mating;
3. Crossing;
4. Mutation;
5. Integration of new individuals into the population;
6. If it was not the last generation, repeat the process from the beginning;
7. Termination of the module, based on the diagram; see Figure 3.

**Figure 4:**
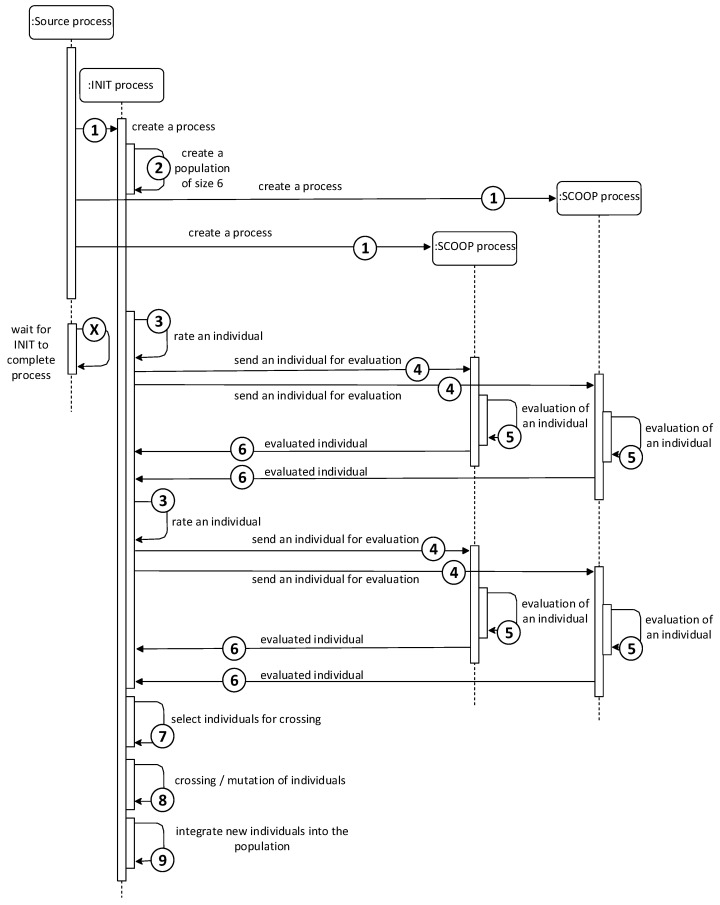
Sequence diagram of one iteration of the Master–Slave model with 2 SCOOP processes.

The first point of the course, the evaluation of individuals, takes place in parallel using SCOOP processes. The futures.map function with parallel processing capabilities was used, which sends the submitted data to other processes for processing. The data of all individuals in the population are thus effectively distributed among all processes for calculating the ability function. The overall start of the module (first iteration) is illustrated by the sequence diagram in Figure 4. According to the diagram, the INIT process is responsible for sending data and, using the futures.map function, is also in charge of the INIT process.

**Figure 5:**
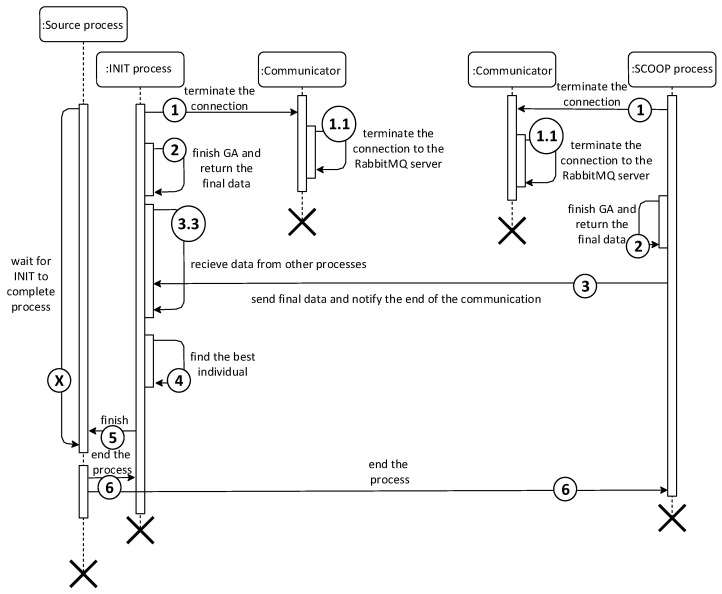
Module termination diagram for the Fine-Grained and Coarse-Grained model.

The main Python class for implementing the Fine-Grained model is the Python class FineGrainedBase. It inherits functionality from the GrainedGeneticAlgorithmBase class. The course of the algorithm no longer copies the course of the classical GA. The GA calculation is divided in parallel among the topology nodes. The course of the Fine-Grained model on one node can be summarized in the following points:

1. Evaluation of the individual (each node works with only one individual);
2. Sending the current individual;
3. Receiving data from nodes (processes) in the neighborhood;
4. Pseudorandom or deterministic selection of one of the received individuals;
5. Crossing and mutation of the current individual with the selected individual;
6. If it was not the last generation, repeat the course from the beginning;
7. Termination of the module, Figure 5.

The implementation of the Coarse-Grained model algorithm is found in the Python class CoarseGrainedBase. As with the Fine-Grained model, it inherits from the GrainedGeneticAlgorithmBase class and modifies the course of the classical GA for processing in each node as follows:

1. Population evaluation (each node works with the whole population);
2. Pseudorandom selection of individuals for mating;
3. Crosses and mutations of individuals;
4. Sending n best individuals to nodes (processes) in the neighborhood;
5. Receiving data from nodes (processes) in the neighborhood;
6. Pseudo-random selection of individuals from the received ones;
7. Integration of selected individuals into the population;
8. If it was not the last generation, repeat the course from the beginning;
9. Selection of the best individual in each node (process);
10. Termination of the module.

The module’s behavior in the case of finding the best solution in the previous generation is identical to the behavior of the Fine-Grained model. Differences from the Coarse-Grained model can be derived from new parameters that do not occur in the Fine-Grained model. The Coarse-Grained model has two variables to represent the population size. One determines the size of the population at the higher level, i.e., at the level of node topology. As with Fine-Grained models, the size is represented in two dimensions, which later define the shape of the neighborhood. The second variable is the size of the population at the level of one node because, in the Coarse-Grained model, each node contains its population.

The second difference is sending individuals between nodes. In the Fine-Grained model, each node sends its individual to the neighboring nodes. Because in the Coarse-Grained model, each node has several individuals within its population, it chooses the best individuals to send. Their number is defined by the user with the num_of_migrants argument.

**Figure 6:**
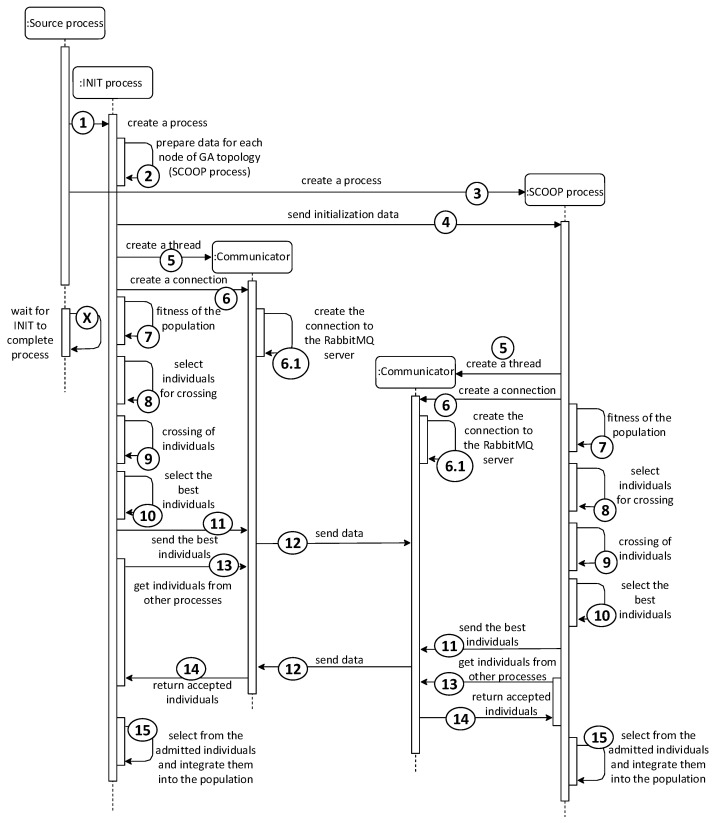
Sequence diagram of one iteration of the Coarse-Grained model for 2 nodes.

The last difference is the initialization of the data for SCOOP processes. Unlike the Fine-Grained model, where one individual is generated for each process, the Coarse-Grained model generates an entire population for each process. The process uses the generated population during the first iteration, within which it subsequently modifies it by mating individuals. The course of the first iteration converted into a sequence diagram can be seen in Figure 6.

## 5. Results and Comparison of the Hardware Utilization of Individuals Models

The verification of GA parallelization’s benefits consisted of comparing the parallel GA models against the Serial one. Since GAs are stochastic, an exact comparison cannot be provided. In this case, a repeated trials statistic was used. In this work, the number of trial repetitions was set to ten. One value was then selected using the median statistical function from the ten results.

The implementation provided process-level parallelization on a single workstation and process-level parallelization on multiple workstations. The comparison of individual GA models was performed on only one workstation to ensure the consistency of the test results. This was due to the different central processing unit (CPU) and memory load of the workstations. Therefore, testing at the multi-workstation level was limited to testing module functionality only, and the actual comparison of individual GA models was performed on a single workstation. The GA parallelization testing was based on parallelizing GA functions that are time consuming.

For the testing, the sphere test objective function (1) was selected.

(1) $$f\left(x\right)=\sum_{i=1}^{D}x_{i}^{2},$$

where the global optima $x_{i}=0,\forall i\in \left\{1\ldots D\right\},\;f\left(x\right)=0$.

The focus of the described research was parallelization possibilities using Python; thus, no complex functions were used. The variable $x_{i}$ in the function can only take bit values, and the variable *D* determines the number of bits. This function was selected from the evolutionary computation program DEAP [20]. The variable *D* was set to 20, so the algorithm minimized the function with values of 20 bit. Thus, the correct result of the minimization should be a zero vector.

The population size gradually increased for each experiment, starting with 64 individuals. For the Serial and Master–Slave GAs, the population size can be arbitrary, but this is not the case for the Fine-Grained and Coarse-Grained GAs. Since the population nodes must communicate with each other in these models, the population size and ordering affect the algorithm’s behavior. For simplicity, a quadratic topology was chosen. Furthermore, masking the space into a toroid shape was performed. Thus, the smallest topology had a size of 8 × 8 (the values refer to the population size) so that even with the smallest size, relative isolation was achieved, and the overlap between neighbors was not too large. Subsequently, the experiments continued with the following topologies: 9 × 9, 10 × 9, 10 × 10, 11 × 11, 12 × 12, 13 × 13, 14 × 14, 15 × 15.

Since modifying the GA behavior for the particular problem to be solved would be too complex and time-consuming, a simplified GA setup was used. The neighborhood size was set to the minimum value of 1. Thus, each neighborhood had a size of 9. The Coarse-Grained model-specific parameter “num of migrants” was also set to the minimum value of 1. The Fine-Grained model-specific parameter “mate best neighboring individual” was turned on so that the algorithm would not introduce randomness into the selection of neighboring offspring.

The testing of the parallel GA models was performed on a Linux server running the Fedora operating system Version 25 and kernel Version 4.11.6-201 virtualizing three other workstations running the Ubuntu operating system 17.10 with kernel Version 4.13.0-16. The physical server contained the Intel(R) Xeon(R) CPU L5408 2.13 GHz processor with four processors. The cache memory size on this processor was 6144 KB. The server also contained 32 GB of RAM (specifically, 32,943,484 KB).

### 5.1. Main Memory Usage Comparison

**Figure 7:**
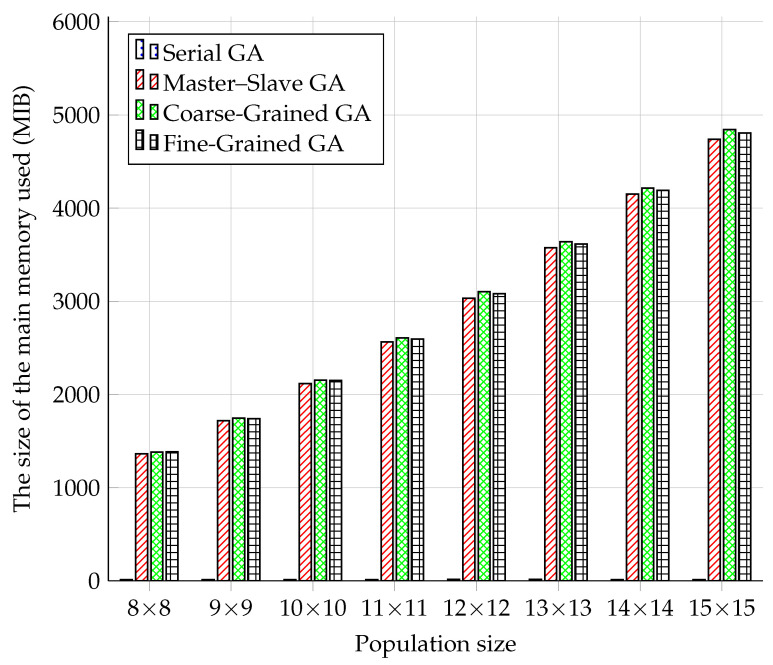
Comparison of GA models in main memory usage.

The results of the measurements in Figure 7 showed that the Serial GA model had, as expected, the lowest demands on the operation memory. In this case, there was no creation of processes using SCOOP and no connection to the RabbitMQ server. With the increasing population size, memory usage did not seem to change. Memory usage decreased at 11 × 11, 14 × 14, and 15 × 15. However, this cannot be attributed to the lower memory consumption of the GA module, but rather to a random decrease in the operating system’s memory usage in the given period of testing.

The results of memory usage measurements were already more evident with parallel GA models. As the size of the population increased, so did the use of memory. For all three parallel models, it can be stated that the increase in memory usage was roughly linear. This was a surprising fact, given that the population increased quadratically.

The Coarse-Grained model had the most significant usage of memory. The second in line was the Fine-Grained model, and the Master–Slave model had the lowest memory usage. However, the differences between the parallel models were minimal. However, the parallel models used several-thousand-times more memory than the Serial model.

### 5.2. CPU Usage Comparison

**Figure 8:**
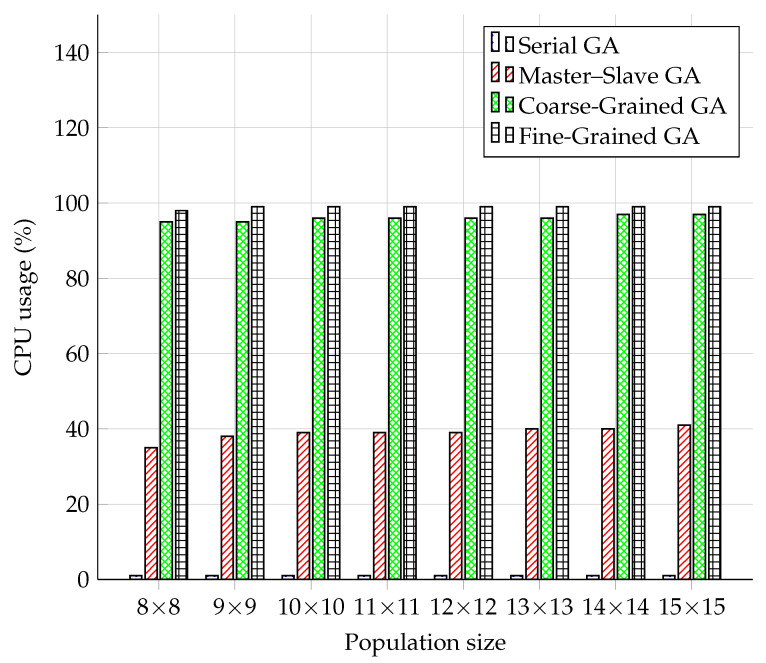
Comparison of GA models by CPU usage.

When comparing memory usage and measuring the CPU usage, it should be noted that the measured results may be skewed due to the running operating system and virtualization used. Even though we used the Ubuntu operating system on all test devices and the same hardware mentioned in Section 5, taking this into account, the differences may be in running processes on the testing device, etc. The results in Figure 8 show a similar scenario in the case of the Serial model as in the memory measurement. The Serial model remained at the level of 1% CPU utilization throughout the course. Compared to other parallel models, the Master–Slave model was more efficient in the case of CPU usage than in the case of memory usage. The Coarse-Grained and Fine-Grained models used the CPU almost 100%, while the Fine-Grained model used about 3% more CPU than the Coarse-Grained one. The course of the CPU usage can hardly be described as linear, although a specific increase in the values was present over time. However, it can be stated that increasing the population size did not have as significant an effect on the CPU as it did on the operation memory.

### 5.3. The Verification of Module Parallelization on Several Workstations

As already mentioned, all measurements were performed on one workstation. Therefore, this part is intended to verify the module’s functionality for parallel GA on several workstations.

**Figure 9:**
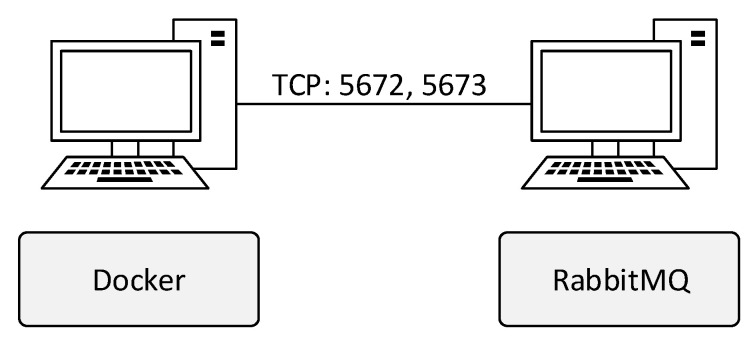
Test environment for several workstations.

According to the test scenario taken from [4] in Figure 9, the environment consisted of workstations created by the Docker platform. The size of the population was chosen to be the smallest possible, i.e., 8 × 8. The functionality of this scenario can be verified by checking the logs from the parallel GA module. The SCOOP module provides its sub-module called the logger for creating records. It was used in every part of the GA module. In this way, records were created not only during the operation of the SCOOP module, but also during the operation of the GA or when sending and receiving data.

By finding the right solution with the chromosome size of 20 bit, all three models of parallel GA on multiple workstations were verified. The Master–Slave model was verified by 7000 iterations, the Fine-Grained model by 70 iterations, and the Coarse-Grained model by 10 iterations. As in the single workstation scenario, a different number of iterations did not degrade the GA’s efficiency. The GA behavior was the same on any number of workstations. However, in the scenario on one workstation, the results were averaged. In the scenario of multiple workstations, it was only a matter of verifying that the module could iterate at high numbers and handle heavy loads.

### 5.4. Individual Models and Comparison of Their Computation Time

The contribution of parallelization generally can be evaluated by comparing the time taken by each compared model to find the best solution. The efficiency of the algorithm itself can be evaluated by comparing the number of iterations. Parallel processing significantly helped to achieve better results. The computing speed of the Master–Slave model depended on how fast the SCOOP model could run its processes and exchange data internally. Another factor in the Fine and Coarse-Grained models was the RabbitMQ server. This server was used for the migration of individuals.

Before comparing the different models, it is helpful to mention the measured delays caused by the SCOOP module. The times in this case and the other cases were read from the module logs. Table 1 shows the start-up delay and delays at the end of the module.

**Figure 10:**
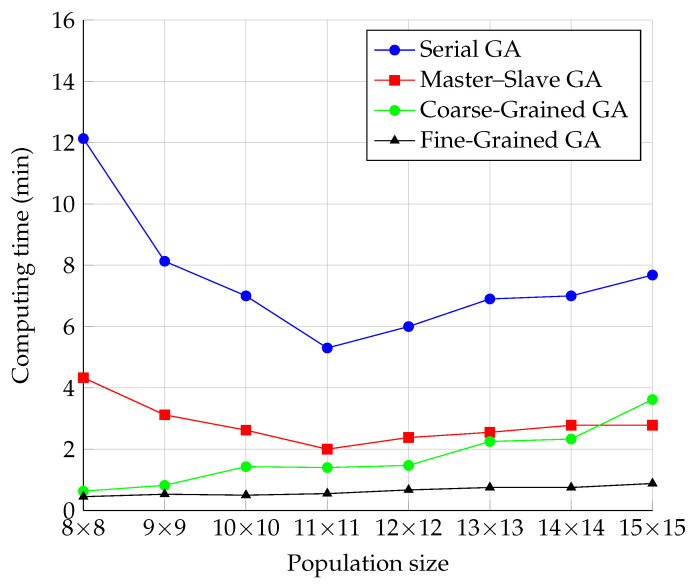
Comparison of the computation time of the GA models.

The results of the measurements of the time required to find the correct solution and their illustrations are shown in Figure 10. To better visualize the comparison, the computational time was converted to minutes. As with the progression of the number of iterations, the similarity between the progressions of the Serial and Master–Slave models can be noticed, as well as the similarity between the Fine-Grained and Coarse-Grained models. The waveforms of the Serial and Master–Slave models were similar to those for the number of iterations. However, the trend was reduced for the dimension 11 × 11 by decreasing time changes. Further, the computational time started to increase with the increasing population size.

In the case of the Fine-Grained and Coarse-Grained models, the time increased with increasing population size from the beginning of the run. This phenomenon can be explained by the fact that the algorithm had to process a larger and larger population, and the increase in this time exceeded the time saved by fewer iterations. This was true for all GA models, including the Serial one. We assumed that the fine-grained model was faster for a sufficient number of processors (independent of population size) in this case, but we needed to prove it by testing it.

For the parallel models, the time consumed by the SCOOP module was added to this. Since the neighborhoods were set constant to a size of nine during testing, the time consumed by communication should be the same for all population sizes. The only delay in communication may have been because the RabbitMQ server’s load increased as the topology increased, and it may have had difficulty processing all requests on time.

### 5.5. The Comparison of the Number of Iterations of Each GA Model

In follow-up testing, we compared the number of iterations for each GA model. The number of iterations of a GA depends on its behavior and the specific parameters of the GA type. Since the behavior of the Master–Slave model was the same as that of the Serial model, the GA parameters were identical for both models. Hence, the statistics of the number of iterations was not performed for each model separately, but only on the Serial model.

**Figure 11:**
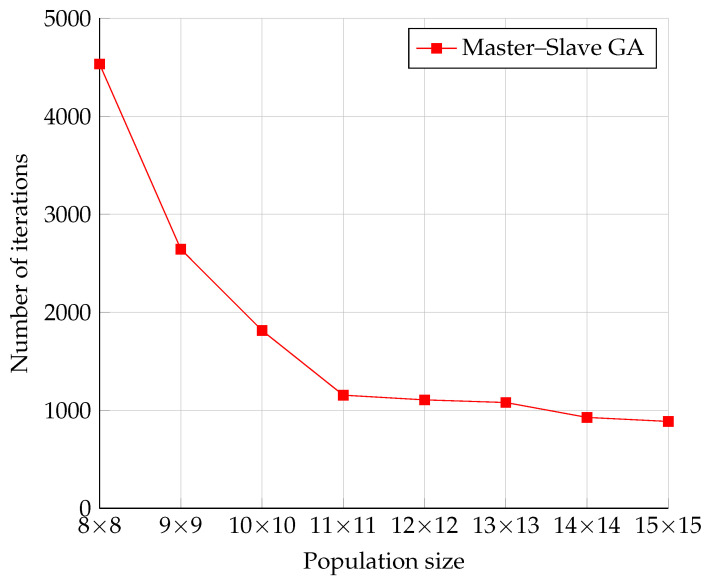
The number of iterations of the Serial and Master–Slave GAs.

The measurement results illustrated in Figure 11 show the expected behavior, namely the decrease in the number of iterations as the population increased. This was a consequence of the previously mentioned fact that a larger population means a broader coverage of the solution space. Thus, there was a higher probability of finding a solution with broader space coverage. The rate of decrease of the number of iterations approached an exponential decrease.

The Fine-Grained and Coarse-Grained models already modified the GA behavior, and therefore, a different behavior was assumed from the Serial and Master–Slave models, respectively. It is worth noting that the Coarse-Grained model’s population dimensions defined the topology dimensions. Meanwhile, each topology node was a separate GA with its population. In testing, the population size of each node was identical to the dimensions of the topology. For example, for the dimension 8 × 8, the Fine-Grained model had a population size of 64 individuals. The Coarse-Grained model, however, had a population size of 64 individuals. Thus, the population at the nodes also increased by increasing the population dimensions in the Coarse-Grained model. In this way, the size of the topology affected the behavior of the nodes among themselves and the behavior of the nodes themselves.

**Figure 12:**
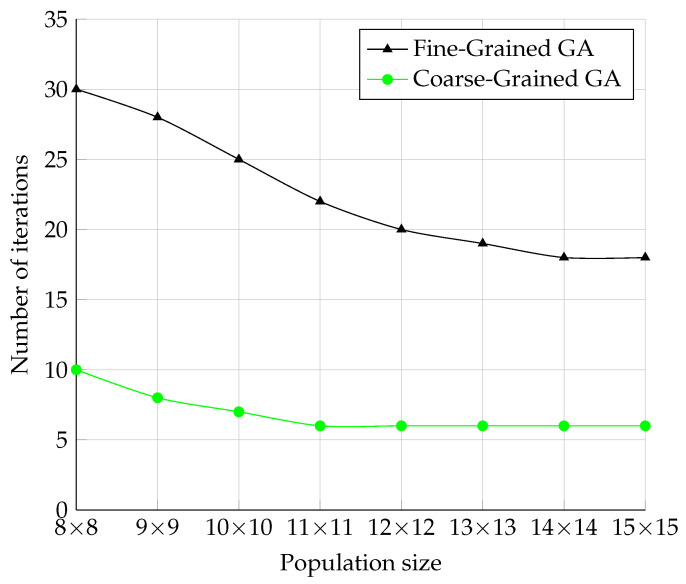
The comparison of Fine-Grained and Coarse-Grained model in the number of iterations.

The measurement results in Figure 12 showed that these two models were much more efficient in terms of the number of iterations, as the number of iterations needed to find the correct solution was roughly 100-times less than in the Serial model. The Coarse-Grained model required even three-times fewer iterations than the Fine-Grained model.

The waveforms of the graphs of both models look roughly the same. This could be because these models have a similar behavior in opposition to the Serial and Master–Slave models, which do not use migration. Another finding was the behavior of the population size. When the population size increased, the number of iterations did not decrease linearly and stayed static. One possible hypothesis to explain this is that both models need a certain number of iterations before changing genes from one node to the other. By increasing the size of the node topology, migration becomes more difficult.

## 6. Conclusions

In this paper, a test scenario was presented together with the environment in which the tests were performed, and finally, the measurement results were presented. The scenario took into account the stochasticity of GAs by repeated measurements. The scenario also defined the most critical parameters to be measured during testing. These were the number of iterations, the computation time, and the CPU, and Random Access Memory (RAM) usage. The mentioned GA models were successfully tested in the scenario with one workstation and also in the scenario with several workstations. Testing on several workstations revealed operating system limitations with the load increases above a specific limit.

The Fine-Grained and Coarse-Grained models were more efficient in the number of iterations because, according to the test results, they needed fewer iterations than the serial model. This may be attributed to the fact, as mentioned earlier, that the Coarse-Grained model contains a separate population at each node and thus covers a much larger solution space.

The number of iterations decreased with increasing the population size in all three GA models. The computational time had a similar course, although in some cases, it increased. However, the parallel GA models saved time compared to the Serial model. The fastest model was the Fine-Grained model. The use of acceleration, efficiency, and scaling parameters for comparison between serial and parallel models yielded further results. On average, the 11 × 11 population size was the fastest for parallel models, and the size 8 × 8 was the most efficient.

The theoretical acceleration for the size 8 × 8 was 64-times. The Master–Slave model achieved almost three-times the acceleration. The Coarse-Grained model approached 20-times the acceleration. The Fine-Grained model achieved the best result with almost 27-times the acceleration compared to the Serial model. The theoretical value for the efficiency parameter was equal to one multiple of the acceleration for the computational unit. The Fine-Grained model with four-tenths of the acceleration for the computational unit came closest to this value: the Coarse-Grained model lagged behind the Fine-Grained model by one-tenth. The Master–Slave model achieved only four-hundredths of the acceleration for the computational unit. As the theoretical values of acceleration and efficiency cannot be achieved in practice, these results were evaluated as positive ones. The last parameter, scalability, was evaluated positively only in the case of the Master–Slave model. The implementation of parallelization generally entails significant hardware requirements. When measuring the algorithm’s parameters, the system parameters were also measured. The measurements were performed using the Linux tool top. It should be noted at the outset that these results may be skewed due to the presence of a running operating system and its associated processes, which also burdened the system.

However, an attempt to minimize possible bias was that both servers had a newly installed operating system, and we formatted the hard drive. It should also be noted that since these were servers, no graphical environment was running in the operating system, which also reduced the load on the system. Finally, it is worth noting that the load in the normal state, i.e., without running the genetic algorithm, was subsequently subtracted from the measured memory and central processing unit usage. The values obtained in this way only reflect the load of the parallel GA module.

As for the module comparison, the advantage of the Multiprocessing module is that it is part of the standard Python libraries. Therefore, there is no need to install third-party modules. However, implementing parallel processing on multiple workstations is more complex than the other modules. The Celery module provides many options and is used by reputable applications such as Instagram, Mozilla addons, and AdRoll [13]. Furthermore, working with the executing worker units is intuitive and straightforward. The drawbacks are the rather complex return of results through various third-party systems and the necessity to run the executing units on each workstation separately. The PyCSP module provides a simple implementation of tasks and communication between them. However, the failure mentioned above to implement the scenario on multiple workstations is a problem. The last module, SCOOP, provides the most straightforward task implementation of the above-mentioned modules. The most significant advantage is the central triggering of task execution on multiple workstations without user intervention on all workstations.

The aim of the research was to compare the performance of different models of parallelized genetic algorithms using SCOOP as the selected Python module. The comparison of the primary memory usage yielded the expected results. As the size of the population increased, so did the memory usage. Surprisingly, the Master–Slave model had similar results as other parallel GA models. However, it did not have to process data received from other processes, as the Master–Slave model does not contain any communication between processes. The Serial model was the least memory intensive, as expected. When the population increased, the CPU usage did not increase as dramatically as the memory usage. Another difference compared to the memory usage was the significantly lower CPU usage of the Master–Slave model compared to other models of parallel GAs. However, the Serial model had the lowest load, as expected.

When parallelizing with multiple workstations, an operating system limitation was observed in some cases. The values of the computational time, number of iterations, and hardware utilization were measured during testing on one workstation. The results confirmed the benefits of parallelization for genetic algorithms, as all three models of the parallel genetic algorithms achieved significant acceleration and efficiency compared to the Serial model.

According to the test results, in terms of performance, concerning the number of iterations, the Fine-Grained and Coarse-Grained models were more efficient because they needed much fewer iterations than the serial model.

In upcoming research, we will focus on the parallelization of genetic algorithms distributed in clusters with the possibility of selective control.

## Figures and Tables

**Table 1:** The delay of parallel GA models caused by parallelization using SCOOP.

Population Size	8 × 8	9 × 9	10 × 10	11 × 11	12 × 12	13 × 13	14 × 14	15 × 15
Start (s)	4	6	7	8.5	10	11.5	13.5	15.5
End (s)	1.5	1.7	2.2	2.5	3.2	3.5	4.1	4.7

## Data Availability

Data are contained within the article.

## Abbreviations

The following abbreviations are used in this manuscript:

AMQP	Advanced Message Queuing Protocol
API	Application Programmable Interface
CPU	Central processing unit
DEAP	Distributed Evolutionary Algorithms in Python
FIFO	First in, first out
GA	Genetic algorithms
GAFT	Genetic Algorithm Framework
IP	Internet Protocol
JSON	JavaScript Object Notation
MPI	Message Passing Interface
NFS	Network File System
OTP	Open Telecom Platform
RAM	Random access memory
RPC	Remote Procedure Call
SCOOP	Scalable Concurrent Operation in Python
SQS	Simple Queue Service
UML	Unified Modeling Language
